# Effective Inhibition of Candidiasis Using an Eco-Friendly Leaf Extract of *Calotropis*-*gigantean*-Mediated Silver Nanoparticles

**DOI:** 10.3390/nano10030422

**Published:** 2020-02-28

**Authors:** Enas M. Ali, Basem M. Abdallah

**Affiliations:** 1 Department of Biological Sciences, College of Science, King Faisal University, Al-Ahsa 31982, Saudi Arabia; eabdelkader@kfu.edu.sa; 2 Department of Botany and Microbiology, Faculty of Science, Cairo University, Cairo 12613, Egypt

**Keywords:** silver nanoparticles, *Calotropis gigantea*, *Candida albicans*, dimorphism, biofilm

## Abstract

The approaches used for the green biosynthesis of nanoparticles with clinical applications have been widely used in nanotechnology due to their potential to provide safe, eco-friendly, cost effective, high-stability, and high-loading-capacity nanoparticles. This study aimed to evaluate the anti-candidal activity of silver nanoparticles (AgNPs) biosynthesized using the aqueous leaf extract of *Calotropis gigantea* (CG) alone or in a combination with the plant extract of CG (AgNPs/CG). AgNPs were characterized using UV-Vis spectrophotometry, Fourier transform infrared spectroscopy (FTIR), transmission electron microscopy (TEM), and X-ray diffraction (XRD). The results of the standard disk diffusion method revealed that AgNPs alone displayed anti-candidal activity (11.33-mm inhibition zone), while AgNPs/CG displayed a strong synergistic anti-candidal activity (17.76-mm inhibition zone). Similarly, AgNPs/CG completely inhibited the growth of *C. albicans* after 4 h of incubation, as measured using the time-kill assay. In addition, AgNPs/CG inhibited the dimorphic transition of *C. albicans* and suppressed both the adhesion and the biofilm formation of *C. albicans* by 41% and 38%, respectively. The treatment of *Candida. albicans* with AgNPs/CG showed a significant inhibition of the production of several antioxidant enzymes. Interestingly, AgNPs/CG did not show any cytotoxicity in animal cells, including the MCF-7 cell line and primary mouse bone marrow-derived mesenchymal stem cells (mBMSCs), at the concentration used to completely inhibit the dimorphic transition of *C. albicans*. In conclusion, we identified AgNPs/CG as a promising natural-product-based nanoparticle that can potentially be used as an anti-candidal drug.

## 1. Introduction

*Candida albicans* is a member of the human gut flora and is also an opportunistic pathogenic yeast [1]. *C. albicans*, together with other species of *Candida*, such as *C. tropicalis*, *C. parapsilosis*, and *C. glabrata*, are responsible for approximately 50–90% of all cases of candidiasis in humans [2]. *C. albicans* is identified as one of the most common agents responsible for invasive candidiasis and its infection causes a mortality rate of 40% for patients with systemic candidiasis. Invasive candidiasis contracted in a hospital setting could cause up to 11,200 deaths annually in the United States [3]. *C. albicans* can be found in the oral cavity of 75% of the population. In immunocompromised individuals, it can cause recalcitrant infections of the oral cavity termed oral candidiasis. Approximately 75% of all women suffer at least once in their lifetime from vulvovaginal candidiasis, with 40%–50% experiencing at least one additional episode of infection [4].

A wide range of virulence factors supports the infection ability of *C. albicans*, including the morphological transition between yeast and hyphal forms (phenotypic switching), and the expression of adhesions and invasions in the cell surface and the secretion of hydrolytic enzymes. The formation of biofilms also plays an important role in pathogenesis, where cells in a biofilm display a higher resistance to antifungal drugs because of the improper penetration of antifungal agents [5]. Consequently, the inhibition of biofilm formation and dimorphic transition are considered effective strategies for controlling the virulence and pathogenicity of *C. albicans* and form the basis for development of anti-fungal drugs [6]. Additionally, the effectiveness of current antifungal drugs is limited due to the emergence of resistant *Candida* biofilms and their toxicities [7].

Recently, great attention has been paid to the production of nanoparticles as an antimicrobial drug to be used either alone or in combination with current traditional drugs. In this context, the antimicrobial action of bio-factorized AgNPs with promising therapeutic effects was reported in many studies [8,9]. The green synthesis of nanoparticles by biological methods using plants has shown numerous advantages, including low costs and the large-scale production of nanoparticles, as well as being environmentally safe [10]. On the other hand, nanoparticle production using physical methods demands high energy consumption and chemical methods produce hazardous and toxic compounds that reduce the biological applications of generated nanoparticles [11]. In this study, we used *C. gigantea* L. (Asclepiadaceae) for the green synthesis of AgNPs due to its multiple reported therapeutic potentials, including anti-inflammatory, analgesic, anticonvulsant, anxiolytic, sedative, antidiarrheal, antipyretic, and antimicrobial effects [12,13]. In addition, leaves and areal parts of the plant were reported to have anti-bacterial, anti-fungal [14], and anti-oxidant activities [15]. Due to its wide range of bioactivities, crude aqueous extracts of *C. gigantea* have previously been used for the green synthesis of nanoparticles, including AgNPs and CuO NPs, which have shown remarkable anti-bacterial activity and applications in dye-sensitized solar cells, respectively [16,17,18]. In this study, we investigated for the first time the anti-candidal activity of combined green synthesized AgNPs with *C. gigantea* plant extracts (AgNPs/CG) against *C. albicans*. Our data demonstrated for the first time the superior anti-microbial potential of AgNPs/CG over AgNPs alone in inhibiting *C. albicans* growth via suppressing the dimorphic transition, anti-oxidant enzymes, and biofilm formation, with no sign of cytotoxicity on cultured animal cells.

## 2. Materials and Methods

### 2.1. Chemicals 

Nutrient agar, sabouraud dextrose agar (SDA), Sabaroud dextrose broth (SDB), RPMI-1640 medium, amphotericine b, dimethyl sulphoxide (DMSO), 3-(4,5-dimethylthiazol-2-yl)-2,5-diphenyltetrazolium bromide (MTT), phenylmethylsulphonyle fluoride (PMSF), sucrose, 1-chloro-2,4-dinitrobenzene (CDNB), glutathione, hydrogen peroxide (H_2_O_2_), were purchased from Sigma-Aldrich Chemical Co. (St. Louis, MO, USA). 

### 2.2. Plant Collection

Leaves of *C. gigantea* were collected from shudqum (Al Hassa–El Dammam Road), Eastern Providence, Saudi Arabia. The plant was classified at the Cairo University herbarium. Herbarium samples (voucher number S6E8) were kept at the Department of Botany and Microbiology, Cairo University. The plants were allowed to air-dry at room temperature, then leaves were ground into powder and stored for further experiments.

### 2.3. Test Organism and Growth Conditions

**Figure 1:**
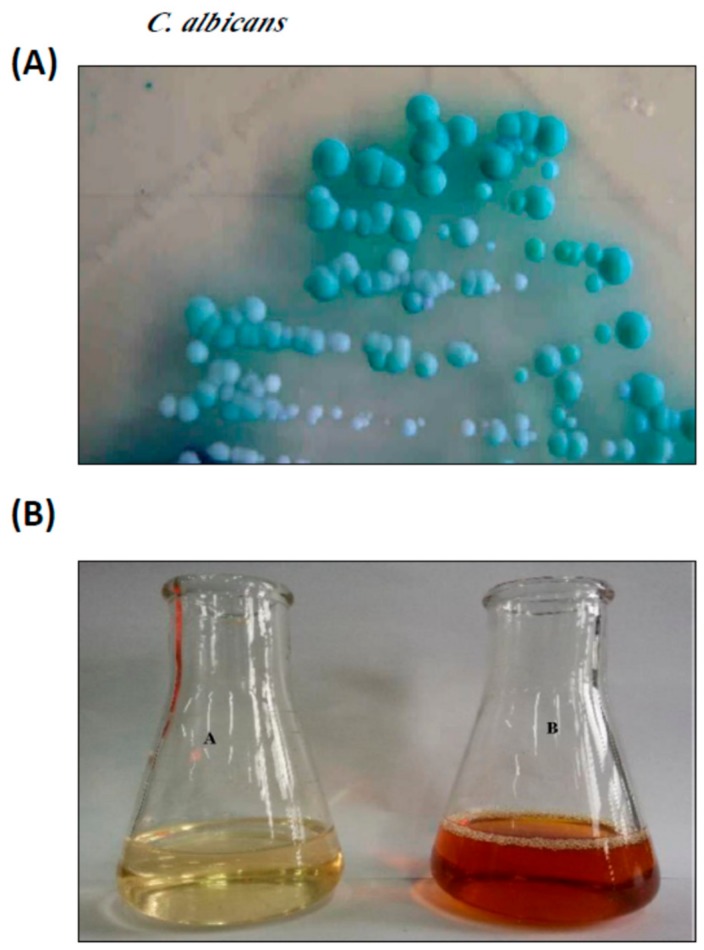
Synthesis of AgNPs from the leaf extract of *Calotropis gigantean* (CG). (**A**) *Candida albicans* isolated from sputum samples, cultured on Sabouraud’s dextrose agar (SDA). (**B**) Silver nitrate solution before (A) and after (B) the synthesis of AgNPs from the leaf extract of *C. gigantean.*

The *C. albicans* strain used in this study was isolated and identified by our group as previously described [19] (Figure 1A), and kept on Sabouraud’s glucose agar slopes at 4 °C. Inoculates were prepared from cultures on Sabouraud’s agar slopes incubated at 37 °C for 16–18 h. The yeast cells were washed in sterile water, centrifuged, and re-suspended in water (under aseptic conditions). The number of blastospores/mL of suspension were determined using hemocytometer counting and a suitable volume of suspension was added to 250 mL Erlenmeyer flasks containing 100 mL of broth to yield an initial concentration of 10^6^/mL blastospores. 

### 2.4. Preparation and Characterization of AgNPs

#### 2.4.1. Preparation of the Leaf Extract

Fresh leaves were washed with distilled water, then cut into small pieces and allowed to dry at room temperature. Ten grams of leaves were boiled in 100 mL distilled water for 20 min and filtered through Whatman No. 1 filter paper [20].

#### 2.4.2. Biosynthesis of AgNPs

An aqueous solution (1 mM) of silver nitrate (AgNO_3_) was mixed with leaf extract. The mixture was kept in a microwave oven at 300 W for 4 min to complete the bio-reduction of AgNO_3_ to Ag+ ions. Complete reduction was confirmed by changing the color from colorless to colloidal brown and saturation was detected using UV-visible spectrophotometric scanning with an Agilent 8453 spectrophotometer (Santa Clara, CA, USA) [20].

#### 2.4.3. Transmission Electron Microscopy (TEM)

TEM was performed according to the method of Teranishi et al. [21] on a Leo 912 AB instrument (Aalen, Germany). Briefly, a drop of diluted sample of AgNPs was poured onto carbon-coated copper grids and allowed to stand for 2 min before imaging. 

#### 2.4.4. X-ray Diffraction Analysis (XRD)

The lyophilized AgNPs coated on an XRD grid were exposed to XRD measurements. The analysis was carried out in an X-ray diffractometer with an operating voltage of 45 kV and a current of 0.8 mA (Unisantis XMD-300, Geneva, Switzerland). The diffraction patterns were found using Cu-Kα radiation of wavelength 1.54 Å in the region of 2θ from 30° to 80° [22].

#### 2.4.5. Fourier Transform Infrared Spectroscopy (FTIR)

The aqueous leaf extract and AgNPs were subjected to FTIR spectroscopy (Thermo Nicolet AVATAR 370, Waltham, MA, USA) to examine their spectra. The analysis was done with KBr pellets, recorded in the range of 500–4000 cm^−1^ [22].

### 2.5. Anti-Candidal Activity 

A disk diffusion method was used to evaluate the anti-candidal activity of AgNPs and AgNPs/CG, as described previously [23]. Prior to use, the solution of AgNPs was prepared via dissolving AgNPs in 5% dimethylsulfoxide (DMSO, 1000 µg/mL). The sample was sonicated for 15 min and sterile filter paper disks containing 50 µg of AgNPs/disk were used for the assay. A standard antifungal agent, amphotericine b, at 5 µg/disk was used as a positive control, while 5% DMSO was used as a negative control. For the preparation of AgNPs/CG: AgNPs (50 µg/mL) and plant extract (200 µg/mL) were mixed and sonicated for 15 min at room temperature. Paper disks were prepared by adding 50 µL of the AgNPs/CG mixture solution to a 6-mm filter paper disk that contained 50 µg AgNPs and 5 µg amphotericin b. The culture of *C. albicans* was diluted to 1 × 10^6^ CFU and the anti-candidal activity of the AgNPs and AgNPs/CG were evaluated by measuring the diameter of inhibition zones after 48 h of incubation at 28 °C. The minimum inhibitory concentration (MIC) of the AgNPs and AgNPs/CG were determined using the bi-fold serial dilution method [24]. Different concentrations of AgNPs and plant extract (6.25–400 µg/mL) were used for the MIC assay. MIC values were expressed as µg/mL.

### 2.6. Time-Kill Assays 

The anti-candidal activities of AgNPs and AgNPs/CG were evaluated at 0, 4, 8, and 10 h using a colony-counting method. At the determined time, an aliquot of 30 μL was taken from each test suspension and inoculated into SDA for quantifying the colony forming units. Sabaroud dextrose broth (SDB) without any antifungal agent was used as a control [25].

### 2.7. Assay of C. albicans Hyphal Development in Liquid Media

Cultures of *C. albicans* grown overnight were inoculated at 37 °C for 24 h with shaking in RPMI-1640 medium (hyphae-inducing media) supplemented with either amphotericin b, AgNPs, or AgNPs/CG. RPMI-1640 medium without any antifungal agent was used as a control. Aliquots of fungal cells were harvested after 24 h and examined using a bright field with Digital Cell Imaging System (Logos Bio Systems, Heidelberg, Germany) [26].

### 2.8. Adhesion and Biofilm Formation Assays

*C. albicans* suspensions (1 × 10^6^ cells/mL) were inoculated in RPMI-1640 medium supplemented with 0.25% glucose and added to 96 microtiter plates (Nunc, Roskilde, Denmark). AgNPs, plant extract, and AgNPs/CG were added separately to cultured *C. albicans* and incubated for 2 h (to measure adhesion) and 24 h (to measure biofilm formation) at 37 °C under static conditions. After incubation, non-adherent cells were removed via washing and the wells were washed. Biofilm growth was measured using an MTT metabolic assay [27]. Wells without any antifungal agent were used as controls, while those without biofilms were the blanks.

### 2.9. Determination of Antioxidant Enzymes

*C. albicans* was grown overnight at 37 °C in the presence of AgNPs/CG. Cells were harvested and homogenized in a homogenizing buffer (1 mmol/L phenylmethylsulphonyle fluoride, 250 mmol/L sucrose, 10 mmol/L Tris–HCl, pH 7.5). The cells were then disrupted at 4 °C using a soniprobe. The homogenate was centrifuged at 20,217× *g* for 1 h at 4 °C. The protein concentration was evaluated using the Lowry method [28] and the antioxidant enzyme activities were determined as follows. Glutathione S-transferase (GST) activity was measured spectrophotometrically through determining the glutathione (GSH) and 1-chloro-2,4-dinitrobenzene (CDNB) conjugates at 340 nm according to the method of Chitme et al. [29]. Catalase (CAT) activity was evaluated by quantifying the decrease in absorbance of hydrogen peroxide at 240 nm according to the method of Kumar et al. [30]. Superoxide dismutase (SOD) activity was measured using the adrenochrome test, which is dependent on the capability of SOD to suppress the autoxidation of epinephrine in alkaline conditions using the method of Lhinhatrakool and Sutthivaiyakit [31]. Glutathione reductase (GSR) activity was evaluated following the method described by [32], where one unit of glutathione reductase activity is known as the amount of the enzyme catalyzing the reduction of 1 μM of NADPH per min. Glucose 6 phosphate dehydrogenase (G6-P) activity was measured by quantifying the reduction of NADP at 340 nm following the previous method of Gupta and Sanjrani [33]. The activity of glutathione peroxidase (GPX) was determined by measuring a decrease in absorbance at 340 nm, suggestive of the disappearance of NADPH as described [32]. The reaction was initiated by the addition of hydrogen peroxide and the enzyme activity was calculated as nanomoles of NADPH oxidized per minute per milligram protein by using a molar extinction coefficient of 6.22 × 103 mol L^−1^ cm^−1^.

### 2.10. Transmission Electron Microscopy

The aliquots from non-treated cells (control) and treated cells were washed, fixed in a solution of 2.5% glutaraldehyde in 0.1 M cacodylate buffer (Sigma), and then washed in 0.1 M cacodylate buffer at pH 7.2. The samples were then post-fixed in 2.0% osmium tetroxide in 0.1 M cacodylate buffer at pH 7. The samples were dehydrated in a series of alcohol and placed in acetone. Infiltration and embedding were done in Epon-812 resin (EMBed-812 Embedding Kit, catalog no. 14120, Electron Microscopy Sciences, Hatfield, PA, USA). Sections were cut with a Porter Blum MT-2 ultra-microtome (Sorval, Liverpool, NY, USA) using glass and diamond blades. Ultrathin sections were compared in a solution of 2.0% uranyl acetate and lead nitrate/acetate. Samples was examined under a JEOL 1200EX II transmission electron microscope (Peabody, MA, USA) [34].

### 2.11. Cell Culture 

Primary mouse BMSCs were isolated from sacrificed 8-week-old male C57BL/6J mice, as previously described [35]. In brief, bone marrow was flushed out from mouse tibia and femurs using BPS and collected in Eppendorf tubes. Bone marrow cells were isolated using centrifugation for 1 min at 400× *g*. Cells were purified via filtration through a 70-μm nylon mesh filter and cultured in 60 cm^2^ flasks in RPMI-1640 medium supplemented with 12% FBS (Gibco Invitrogen, Dreieich, Germany), 1% penicillin/streptomycin (P/S) (Gibco Invitrogen), and 12 μM L-glutamine (Gibco Invitrogen) in a 5% CO_2_ incubator at 37 °C. The non-adherent cells were removed after 6 h and cultured in fresh medium. The medium was changed every 3–4 days and BMSCs were maintained to be subcultured at a split ratio of 1:2.

A human breast adenocarcinoma MCF-7 cell line was obtained from Leibniz Institute DSMZ-German Collection of Microorganisms and Cell Cultures (ACC 115, Braunschweig, Germany) [36]. Cells were cultured in DMEM supplemented with 10% fetal bovine serum (FBS), 1% penicillin/streptomycin (P/S), and 10 µg/mL insulin (Gibco Invitrogen).

### 2.12. Cytotoxicity Assay

The cytotoxicity of different compounds was determined by measuring the cell viability using an MTT cell proliferation assay kit (Sigma-Aldrich) according to the manufacturer’s instructions. The cells were seeded in 96-well plates and treated with different concentrations of either AgNPs or AgNPs/CG for 48 h. The cells were then washed with PBS and incubated with fresh medium containing 0.5 mg/mL MTT to metabolize to formazan. The optical density was determined at 550 nm using an ELISA plate reader [37]. Cell viability was represented as a percentage of the control non-treated cells.

### 2.13. Statistical Analysis

All values are represented as mean ± SD, of at least three independent experiments. A power calculation was performed for two-samples using an unpaired Student’s *t*-test (two-tailed) assuming equal variation in the two groups. Differences were considered statistically significant at * *p* < 0.05, and ** *p* < 0.005.

## 3. Results

### 3.1. Preparation and Characterization of AgNPs

**Figure 2:**
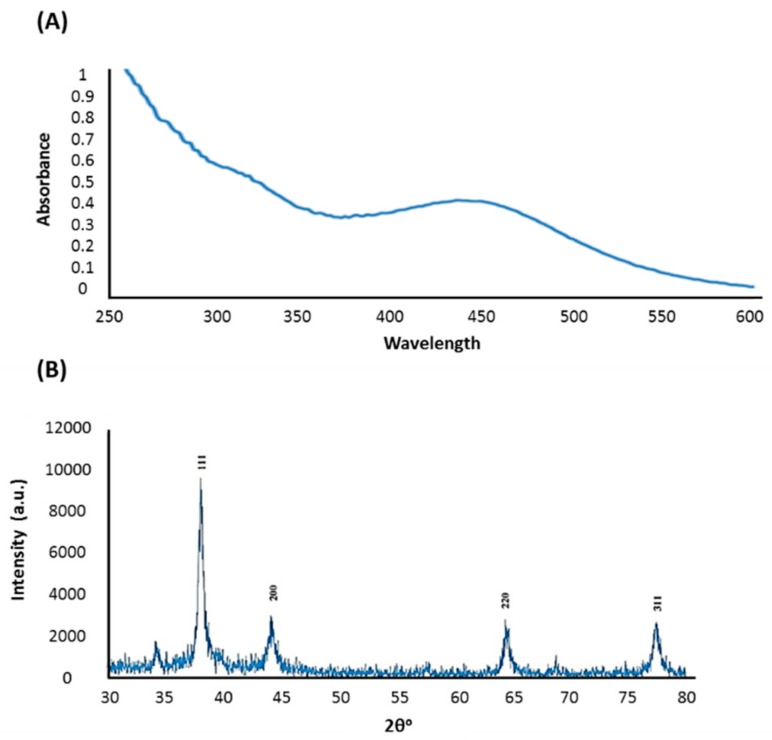
Characterization of AgNPs synthesized by *C. gigantean* in aqueous solution. (**A**) UV-vis absorption spectrum of synthesized AgNPs by a *C. gigantean* leaf extract incubated with silver nitrate (1 mM). The peak values are for the UV-vis plotted between AgNPs/absorbance ratios. The highest absorbance peak was at about 450 nm, corresponding to the plasmon resonance of AgNPs. (**B**) XRD spectrum recorded for AgNPs showed four distinct diffraction peaks at 38.18°, 44.35°, 64.4°, and 77.3° indexed 20(degree) values of (111), (200), (220), and (311) crystalline planes of cubic Ag.

**Figure 3:**
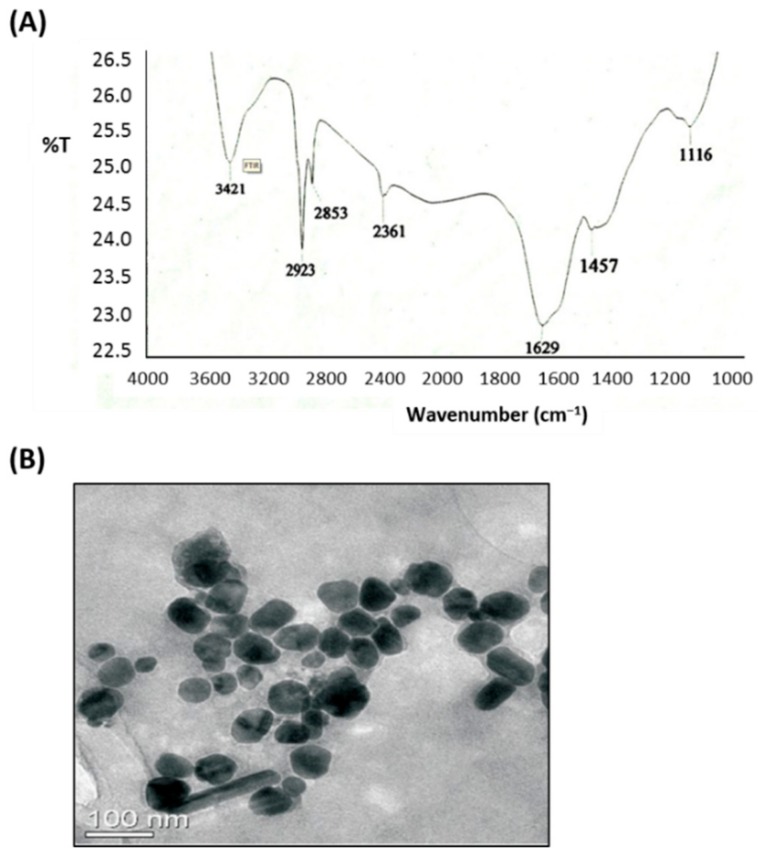
FTIR spectrum and TEM of AgNPs synthesized by *C. gigantean*. (**A**) The FTIR spectrum of the synthesized AgNPs from *C. gigantean* leaf showing seven distinct peaks at 1116, 1457, 1629, 2361, 2853, 2923, and 3421 cm^−1^, revealing the existence of amide, amino, and alkane groups. This proved the availability of abundant chemical constituents that had bound onto the synthesized AgNPs. (**B**) A TEM image showing the spherical shape nanoparticles, where the size was in the range of ≈10–70 nm. The AgNPs were distributed uniformly without significant agglomeration.

In the present study, aqueous leaf extract of *C. gigantea*, a traditional medicinal plant, was used as a reducing and stabilizing agent for the green synthesis of AgNPs. The ability of the bioactive components of *C. gigantea* to act as biocatalysts for the reduction of Ag^+^ to Ag^0^ was evaluated. The change of color of the biomass filtrate after adding AgNO_3_ as a precursor from colorless to yellowish brown was observed (Figure 1B). The UV-visible spectra of biosynthesized AgNPs showed an absorption band peaking at 450 nm (Figure 2A). The color of the solution supports the absorption wavelength in the visible region. The XRD pattern showed clear diffraction line at low angles (30°–80°). The Bragg reflections at angles 2θ of 38.18°, 44.35°, 64.4°, and 77.3° corresponded to the 111, 200, 220, and 311 bands, respectively (Figure 2B). This pattern verified the structure of AgNPs as a face-centered cubic structure. XRD results confirmed the crystal structure of the silver in the *C. gigantean* extract. The FTIR spectrum of biosynthesized AgNPs displayed seven different peaks: 1116, 1457, 1629, 2361, 2853, 2923, and 3421 cm^−1^ (Figure 3A). The peaks at 3421cm^−1^ and 2923 cm^−1^ were due to the NH stretch vibration of the primary and secondary amides of protein. The peak at 2853 cm^−1^ was due to the C–H symmetrical stretch vibration of alkanes. The peak at 2361 cm^−1^ was due to the primary amine group of proteins. The peaks at 1457 cm^−1^ was due to the amino and amino-methyl stretching groups of proteins. The peak at 1116 cm^−1^ was due to the C–O stretching vibrations mode. Finally, TEM gave additional information about the morphology, size, and distribution profile of the AgNPs. The results obtained from the TEM demonstrated that the biosynthesized AgNPs were spherical in shape and the size was in the range of ≈10–70 nm (Figure 3B). In addition, AgNPs were distributed uniformly without significant agglomeration.

### 3.2. Anti-Candidal Activity of AgNPs

**Figure 4:**
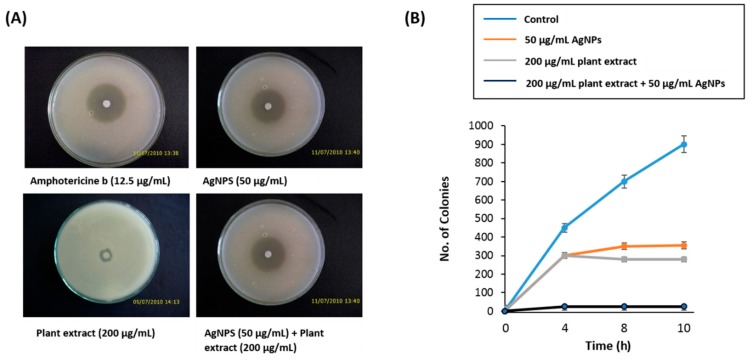
Antimicrobial potential of AgNPs. (**A**) Disc diffusion method showing the anti-candidal activity of AgNPs and the synergistic anti-candidal potential of AgNPs (50 µg/mL) mixed with *C. gigantea* leaf extract (200 µg/mL). Data are expressed as the mean zone of inhibition in mm. (**B**) Time-kill curves of *C. albicans* following exposure to AgNPs, plant extract, and AgNPs/CG. Non-treated *Candida* cells were used as a control. There was a decrease in the rate of cell growth when *Candida* cells were treated with AgNPs/CG compared to either the control cells, AgNPs alone, or plant extract alone. Values are the mean ± SD of three independent experiments.

We studied the anti-candidal activity of AgNPs, *C. gigantea* plant extract, and AgNPs/CG using a disk diffusion method assay. In this assay, the commercially available amphotericin b showed a higher inhibitory effect against *C. albicans* with an inhibition zone diameter of 19 mm. The AgNPs showed a moderate anti-candidal activity with inhibition zone diameter of 11.33 mm, while the plant extract displayed a lower anti-candidal activity with an inhibition zone diameter of 6.1 mm (Table 1 and Figure 4A). The MIC values of AgNPs and plant extract were 50 and 200 µg/mL, respectively (Table 1). 

### 3.3. Synergistic Anti-Candidal Activity of AgNPs and Plant Extract

When AgNPs were combined with the plant extract, they exhibited a synergistic anti-candidal action against *C. albicans*, with a zone of inhibition of 17.22 mm (Table 1 and Figure 4A). In addition, the time-kill curves measured the fungi-static effect for both AgNPs and plant extract at 50 μg/mL and 200 μg/mL, respectively, on the growth of *Candida* cells (Figure 4B). After only 4 h of incubation, AgNPs/CG inhibited the growth of *C. albicans* to zero colonies in contrast to the plant extract alone or AgNPs alone samples, which still had live colonies (Figure 4B). Thus, AgNPs/CG displayed a higher anti-candidal activity than either AgNPs alone or plant extract alone.

### 3.4. Effect of AgNPs/CG on the Virulence Factors of C. albicans 

**Figure 5:**
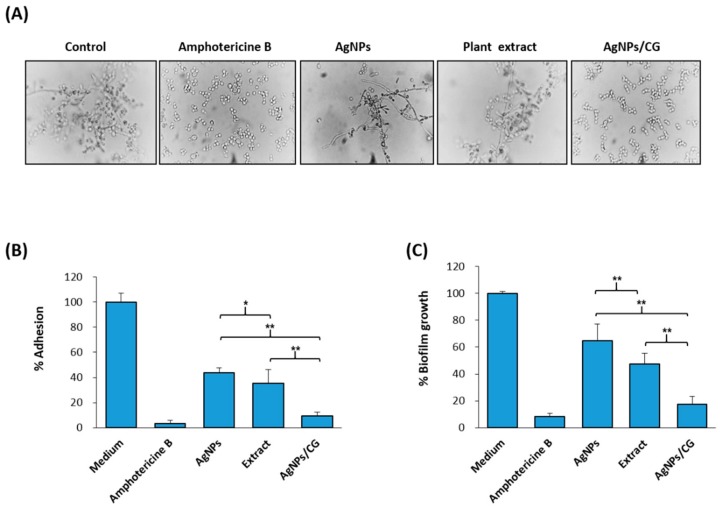
Effect of AgNPs on virulence factors of *C. albicans.* (**A**) Microscopic examination of the dimorphic transition in RPMI-1640 medium supplemented with either amphotericine b, AgNPs, or AgNPs/CG. Complete inhibition of the dimorphic transition (all cells were observed in yeast form when AgNPs and plant extract were combined, unlike when the two were tested on their own). (**B**) Percentage of adhesion after (2 h) in RPMI-1640 medium supplemented with 0.25% glucose. (**C**) Biofilm formation (after 24 h) as measured using an MTT metabolic assay. Values are the mean ± SD of three independent experiments (* *p* < 0.05, ** *p* < 0.005).

As shown in Figure 5A, the untreated controls displayed great hyphal growth after 6 h, while the capacity of *C*. *albicans* to transform morphologically was repressed by AgNPs at concentrations lower than the MIC. On the other hand, hyphal growth was totally blocked in the presence of AgNPs/CG (50 μg/mL AgNPs + 200 μg/mL plant extract), as assessed using microscopic observations. In addition, we examined the anti-adhesive and anti-biofilm efficiencies of the AgNPs, plant extract, or AgNPs/CG. As shown in Figure 5B,C, AgNPs (50 μg/mL) inhibited the adhesion and the biofilm growth of *C. albicans* by 59% and 53%, respectively, while AgNPs/CG inhibited the adhesion and biofilm formation of *C*. *albicans* by 91% and 87%, respectively (Figure 5B,C). 

### 3.5. AgNPs/CG Suppressed the Production of Antioxidant Enzymes by C. albicans

Since, very little is known about the inhibition of the production of oxidative enzymes by nanoparticles, we measured the effect of AgNPs/CG on the production of antioxidant enzymes by *C. albicans*. Interestingly, AgNPs/CG showed the ability to significantly inhibit the following anti-oxidant-related enzymes, including GST, CAT, SOD, G6-P, GSR, and GPX by 85.73%, 86.92%, 83.33%, 87.50%, 85.71%, and 100% respectively in *C. albicans* (Table 2). 

### 3.6. Morphological and Ultrastructural Alteration Caused by AgNPs/CG 

**Figure 6:**
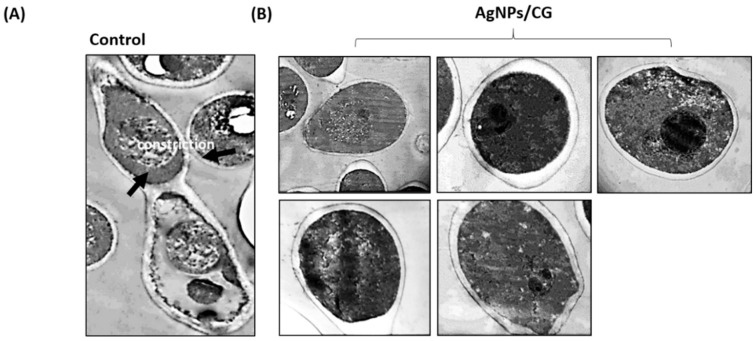
Ultrastructure alteration in *C. albicans* by AgNPs/CG. TEM images of *C. albicans* cells treated with AgNPs. (**A**) Yeasts without treatment showing the typical filamentous morphology of *C. albicans*, which was very elongated but narrow in width (0.6–1.1 µm). (**B**) Yeast after treatment showing the typical *C. albicans* yeast form morphology, where the ovoid cell was surrounded by a cell wall after 24 h of incubation and treatment with AgNPs/CG (50 µg/mL AgNPs + 200 µg/mL plant extract).

We further examined the effect of AgNPs/CG on the ultrastructural alteration of *C. albicans* using TEM. Non-treated cells showed the typical filamentous morphology of *C. albicans*, which was very elongated but narrow in width (0.6–1.1 µm) (Figure 6A). A septum was formed between new filamentous growth and the parent cell, and the cell wall thickness was thinner than that of the yeast-phase cells. In contrast, cells treated with AgNPs/CG showed the typical *C. albicans* yeast form morphology, where the ovoid cell was surrounded by a cell wall. Within the cytoplasmic area, slightly irregular organelles (such as nucleus, mitochondria, endoplasmic reticulum, and nuclei) were found to be frequently ovoid, and a double-unit membrane enclosed them. Mitochondria were elongated (Figure 6B). 

### 3.7. Cytotoxicity of AgNPs/CG

**Figure 7:**
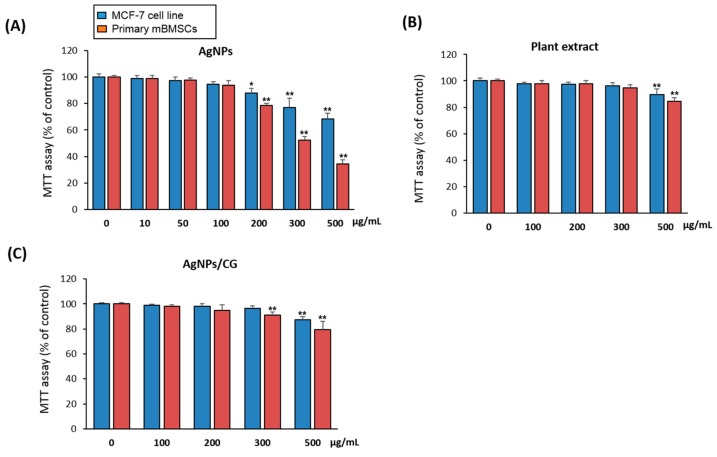
Cytotoxicity of AgNPs, plant extract and AgNPs/CG on animal cell culture. MCF-7 and mBMSCs cells were treated with different concentrations of (**A**) AgNPs, (**B**) plant extract, or (**C**) AgNPs/CG (50 µg/mL + different concentrations of *C. gigantean*) for 24 h, where cytotoxicity was measured using an MTT assay. Values are the mean ± SD (error bars) of three independent experiments (* *p* < 0.05, ** *p* < 0.005 compared to the control non-treated cell line).

As a preliminary step to examine the therapeutic effect of AgNPs/CG in vivo, we examined the cytotoxicity of AgNPs/CG on the human cancer cell line MCF-7 and primary mouse BMSCs using a cell viability MTT assay. As demonstrated in Figure 7A,B, AgNPs showed a cytotoxicity at concentrations above 100 µg/mL, while the plant extract was not toxic to the cells up to 300 µg/mL. Interestingly, AgNPs/CG displayed cytotoxicity at concentration above 200 µg/mL plant extract + 50 µg/mL AgNPs and started to be toxic for both cell types at 500 µg/mL plant extract + 50 µg/mL AgNPs (Figure 7C). 

## 4. Discussion

The biosynthesis of nanoparticles by plants could be a better candidate for the low-cost and large-scale production of nanoparticles due to it being environmentally safe [38]. *C. gigantea* is a common medicinal plant reported as having anti-candidal activity, cytotoxic activity, antipyretic activity, and wound healing activity [14,39]. Numerous compounds have been extracted from *C. gigantea*, such as cardenolides [40], flavonoids [41], and giganticine [42]. In our study, the appearance of a brown color in the reaction solution was a clear indication of the formation of AgNPs in the reaction mixture, as described previously [43,44]. The characterization of the green synthesized AgNPs using the aqueous leaf extract of *C. gigantea* was achieved using techniques such as UV-vis spectroscopy, FTIR analysis, TEM, and XRD analysis [45,46]. The surface plasmon resonance phenomena (SPR) absorbance is sensitive to the shape, size, and nature of particles present in the solution, and also depends upon the inner particle distance and the surrounding media. Spectral analysis revealed the SPR absorption of our green synthesized AgNPs at 450 nm, which was within the typical wavelengths (from 400 to 480 nm) reported for AgNPs [47,48]. 

Our data identified the MIC for AgNPs to be 0.05 mg/mL with a high anti-candidal activity. AgNPs produced using different methods display variable levels of antifungal action based on their size, shape, and surface modification [49,50,51,52]. In this regard, several studies investigated the antimicrobial activity of AgNPs. For example, synthesized AgNPs using the seed extract of *Syzygium cumini* displayed anti-candidal activity at 0.125–0.250 mg/mL [8], while AgNPs biosynthesized using *Artemisia annua* leaf extracts displayed fungicidal action against three clinically important *Candida* species at 120 mg/mL [53].

Several mechanisms were reported to explain the mode of anti-candidal activity of AgNPs. These include the capacity of AgNPs to damage the membrane permeability barrier and to destruct the membrane lipid bilayers, resulting in the leakage of ions, along with forming pores and dissipating the electrical potential of the membrane. In addition, AgNPs were shown to block the cell cycle at G2/M phase in *C. albicans* [54], to increase the production of reactive oxygen species (ROS), and to decrease the activity of metal-based antioxidant enzymes [55].

In this study, we demonstrated that the efficiency of the antifungal activity of AgNPs could be enhanced via the combination therapy of AgNPs with natural antifungal agents to provide a novel strategy for the efficient control of *C. albicans*. To our knowledge, this was the first report to identify the MIC value of a combination between a plant extract and its biosynthesized AgNPs against *C. albicans*. Previously, the anti-candidal action of AgNPs was found to be improved using a combination with commercially available amphotericin b against five *Candida* species [23]. Thus, our data identified *C. gigantea* leaf extract as a source of bioactive antifungal compounds that could contribute in the extracellular synthesis of AgNPs, and has a stronger antifungal action than the previously designated antifungal effect [14,56,57]. 

The phenotypic switching between yeast and hyphal forms has been considered as one of the most significant virulent factors in *C. albicans* [58]. The development of hyphae is an interesting property of *C. albicans* that plays a vital role in adherence and biofilm formation, which is certainly crucial for colonization and pathogenesis [59,60]. The blocking of morphogenesis from yeast to a filamentous form would mean preventing the infection. Our data demonstrated the blocking of hyphal growth using AgNPs/CG. Similarly, several studies demonstrated the suppression of the *C*. *albicans* morphogenesis using AgNPs [6,8,53,61] in a mechanism that involved targeting the Ras-mediated signal transduction pathways in *C. albicans* through the downregulation of the expression of cell elongation gene (*Ece1*), hyphal inducer gene (*Tec*), and yeast to hyphal transition genes (*Tup1* and *Rfg1*), which are key genes for the morphological transition [62]. 

The prevention of blastospore adhesion and differentiation into a filamentous form using AgNPs appears to provide an excellent therapeutic choice [51,63,64,65]. Our results showed that AgNPs/CG was more efficient at suppressing the adhesion and biofilm formation of *C. albicans* than plant extract or AgNPs alone. Similar to our finding, several other studies demonstrated the inhibitory effect of AgNPs on *C. albicans* biofilm formation [8,61,66]. In addition, AgNPs biosynthesized by *Dodonaea viscosa* and *Hyptis suoveolens* leaf extracts were shown to suppress the biofilm formation of *Candida spp.* from 79% to 88% at 10 μg/mL [51]. 

The mechanism underlying the suppression of biofilm formation using AgNPs was reported to include the anti-adhesive action of AgNPs that regulate the growth of living microbial cells and the suppression of microbial adhesions gene expression [27,67,68]. In addition, AgNPs were shown to suppress blastospores and to disrupt the cell wall of both the yeast and the filamentous forms in order to cause the inhibition of biofilm formation in *Candida* [61,66]. 

*C. albicans* has developed enzymatic antioxidant defense mechanisms to reduce the harmful effects of ROS produced by phagocytes during the infection process. Our data demonstrated the inhibitory effect of AgNPs/CG on the production of antioxidant enzymes by *C. albicans*. Supporting our data, *C. gigantea* was found to exert an antioxidant activity in other studies [69,70,71]. AgNPs were also reported to display antioxidant activity via stimulating *C. albicans* to express genes encoding for antioxidants, such as catalase, glutathione peroxidase, superoxide dismutase, and components of the glutathione/glutaredoxin and thioredoxin systems [55,72]. The stimulatory effect of AgNPs on oxidative stress as a mechanism of toxicity in *C. albicans* resulted in the shifting of the total redox balance to oxidation and thus causing the functional destruction of cells [73,74,75]. 

Our data demonstrated the safety of using AgNPs/CG at the concentration of (200 µg/mL plant extract + 50 µg/mL AgNPs) in both primary and transformed cultured animal cells, suggesting the plausible application of AgNPs/CG in vivo in a preclinical study. The toxicity of AgNPs to human cells was found to be attributed to the type of the reducing agent used in the biosynthesis [76]. In this regard, the plant extracts could act as a stabilizing agent to stabilize the particles of AgNPs against dissolution, subsequently reducing the toxicity of AgNPs. In addition, the antioxidant and the anti-inflammatory activities of *C. gigantean* extracts might contribute toward reducing the cytotoxicity of AgNPs [69,70,71]. 

Thus, the combination of AgNPs and *C. gigantean* extracts provide a novel strategy for developing biosynthesized-based drug for preventing candidiasis. 

## 5. Conclusions

In this study, we biosynthesized AgNPs by using the aqueous leaf extract of *C. gigantean*. Our data demonstrated the efficient anti-candidal activity of a combination of biosynthesized AgNPs and the plant extracts of *C. gigantean* (AgNPs/CG). AgNPs/CG significantly inhibited the growth, morphogenesis, adhesion, biofilm formation, and the secretion of antioxidant defense enzymes by *C. albicans*. In addition, AgNPs/CG showed no sign of cytotoxicity, even at concentrations higher than the calculated MIC. Thus, AgNPs/CG provides a novel strategy for preventing the pathogenesis of *C. albicans* by suppressing the key virulence factors and development of biofilms. However, further preclinical studies are needed to evaluate the therapeutic potential of AgNPs/CG in the treatment of candidiasis in vivo.

## Figures and Tables

**Table 1:** Anti-candidal activity of AgNPs and plant extract against *C. albicans.*

Concentration (µg/mL)	Antifungal Agents		
	Amphotericine B	AgNPs	Plant Extract
	IZD (mm)		
0	0 ^a^ ± 0.2	0 ^a^ ± 1.0	0 ^a^ ± 0.2
6.25	19 ^c^ ± 0.2	6 ^b^ ± 1.2	3.6 ^a^ ± 0.8
12.5	No growth	8.22 ^b^ ± 0.5	4.1 ^a^ ± 1.0
25		11.33 ^b^ ± 0.9	4.9 ^a^ ± 1.5
50		No growth	5.2 ^a^ ± 1.5
100			6.1 ^a^ ± 0.9
200			No growth
400			

IZD: Inhibition zone diameter (mm). Data are expressed as the mean zone of inhibition in mm followed by SD. The values with different superscript letters (a, b and c) in the same row are significantly different according to ANOVA and Duncan’s multiple range tests.

**Table 2:** Specific activities of antioxidant enzymes.

Enzyme	Substrate	Specific Activity (U/mg Protein)	
		Control	AgNPs/CG
Glutathone-S transferase	CDNB	0.422 ± 0.11	0.0602
Catalase	H_2_O_2_	2.3 ± 0.33	0.75
Superoxide dismutase	Epinephrine	0.405 ± 0.06	0.0675
Glucose 6 phosphate dehydrogenase	NADP	425.22 ± 5.11	53.15
Glutathione reductase	NADPH	45.23 ± 2.13	6.46
Glutathione peroxidase	NADPH	0.00055 ± 0.0001	0

## Data Availability

All materials are available from the corresponding author.
